# Recent developments in *MrBUMP*: better search-model preparation, graphical interaction with search models, and solution improvement and assessment

**DOI:** 10.1107/S2059798318003455

**Published:** 2018-03-06

**Authors:** Ronan M. Keegan, Stuart J. McNicholas, Jens M. H. Thomas, Adam J. Simpkin, Felix Simkovic, Ville Uski, Charles C. Ballard, Martyn D. Winn, Keith S. Wilson, Daniel J. Rigden

**Affiliations:** a CCP4, Research Complex at Harwell, Rutherford Appleton Laboratory, Harwell Oxford, Didcot OX11 0FA, England; b Institute of Integrative Biology, University of Liverpool, Liverpool L69 7ZB, England; c STFC, Rutherford Appleton Laboratory, Harwell Oxford, Didcot OX11 0FA, England; d York Structural Biology Laboratory, Department of Chemistry, University of York, York YO10 5DD, England; e Synchrotron SOLEIL, L’Orme des Merisiers, Saint Aubin, BP 48, 91192 Gif-sur-Yvette, France

**Keywords:** *MrBUMP*, molecular replacement, automated structure solution, *CCP*4 pipeline

## Abstract

Increasing sophistication in molecular-replacement (MR) software and the rapid expansion of the PDB in recent years have allowed the technique to become the dominant method for determining the phases of a target structure in macromolecular X-ray crystallography. In addition, improvements in bioinformatic techniques for finding suitable homologous structures for use as MR search models, combined with developments in refinement and model-building techniques, have pushed the applicability of MR to lower sequence identities and made weak MR solutions more amenable to refinement and improvement. *MrBUMP* is a *CCP*4 pipeline which automates all stages of the MR procedure. Its scope covers everything from the sourcing and preparation of suitable search models right through to rebuilding of the positioned search model. Recent improvements to the pipeline include the adoption of more sensitive bioinformatic tools for sourcing search models, enhanced model-preparation techniques including better ensembling of homologues, and the use of phase improvement and model building on the resulting solution. The pipeline has also been deployed as an online service through *CCP*4 *online*, which allows its users to exploit large bioinformatic databases and coarse-grained parallelism to speed up the determination of a possible solution. Finally, the molecular-graphics application *CCP*4*mg* has been combined with *MrBUMP* to provide an interactive visual aid to the user during the process of selecting and manipulating search models for use in MR. Here, these developments in *MrBUMP* are described with a case study to explore how some of the enhancements to the pipeline and to *CCP*4*mg* can help to solve a difficult case.

## Abstract 2

New developments in the *MrBUMP* pipeline, including graphical interaction with search models using *CCP*4*mg* and the use of ensemble search models, are reported.

## 1. Introduction   

Molecular replacement (MR) is one of the key methods used to solve the phase problem in macromolecular crystallography (MX). Typically, MR exploits the fact that evolutionarily related macromolecules generally have similar structures. Therefore, when correctly placed in the unit cell of the target structure, a homologous structure can provide a sufficiently correct approximation to the phases of the target. When combined with the amplitude information provided by the X-ray diffraction intensities, these phases enable the determination of the structure factors and hence the electron-density map of the target molecule(s). A detailed introduction to molecular replacement can be found in Evans & McCoy (2008 ▸). Many other papers discussing case studies and developments in the area can be found in the proceedings of the 2013 CCP4 Study Weekend (the November 2013 issue of *Acta Crystallographica Section D*).

At its heart, MR involves a six-dimensional search, but to speed up the process most modern programs break the search into two three-dimensional searches over three angles in rotational space and three directions in translational space. *Phaser* (McCoy *et al.*, 2007 ▸) and *MOLREP* (Vagin & Teplyakov, 2010 ▸) are two of the main MR applications provided by *CCP*4 (Winn *et al.*, 2011 ▸), and both implement this approach. Given a suitable search model, both are highly effective at correctly placing the search model, from which the initial approximate phases for the target can be derived. For proteins, an amino-acid sequence identity of 30% or more between the target and the search model typically indicates sufficient structural similarity for the method to work. Complications can arise owing to problematic data (twinning, anisotropy, pseudo-translation *etc.*) and/or differences between the search model and the target (hinge motion between domains, alternate conformations for side chains and loops *etc*.). Accounting for these complications can assist in obtaining a correct solution. The maximum-likelihood method used in *Phaser* (McCoy, 2004 ▸) is particularly effective when these issues arise, but both *Phaser* and *MOLREP* ultimately rely on the provision of a suitable search model.

Recent work by McCoy *et al.* (2017 ▸) investigated what constitutes a suitable search model based on factors including the resolution of the observed intensities, their measurement error and the expected similarity of the search model to the target. From these factors, they derive an expected log-likelihood gain (eLLG), which is an indicator of the likelihood of a search model being successful in MR. With sufficiently good resolution (1 Å or better) they demonstrated that it is possible to use a single atom as a search model with success. For more modest resolutions, typically between 1 and 2.5 Å, applications such as *AMPLE* (Bibby *et al.*, 2012 ▸) and *ARCIMBOLDO* (Rodríguez *et al.*, 2009 ▸) have shown that it is feasible to use small fragments such as helices as successful search models. At resolutions poorer than 2.5 Å, search models more similar in size to that of the target structure are usually required. In light of this, identifying and preparing search models is a key step that can draw upon the rapidly developing tools in the field of bio­informatics as well as the structural information present in the more than 130 000 depositions (at the time of writing) in the PDB (Berman *et al.*, 2003 ▸; https://www.wwpdb.org/).

Several projects designed to automate the conventional approach to MR have been developed in recent years, including *MrBUMP* (Keegan & Winn, 2007 ▸, 2008 ▸; Keegan *et al.*, 2011 ▸), *BALBES* (Long *et al.*, 2008 ▸), *MRage* (Bunkóczi *et al.*, 2013 ▸) and more recently *MoRDa* (Vagin & Lebedev, 2015 ▸). All implement an approach which automatically searches for and prepares search models for MR, carries out MR using these models and performs some additional post-MR refinement and model rebuilding to improve upon and assess the MR solution. Some unconventional approaches have been developed to tackle cases where only distant homologues or no known homologues are available for use as search models. *AMPLE* (Bibby *et al.*, 2012 ▸; Keegan *et al.*, 2015 ▸; Thomas *et al.*, 2015 ▸, 2017 ▸) implements an approach which uses *ab initio* modelling applications such as *ROSETTA* (Shortle *et al.*, 1998 ▸) and *QUARK* (Xu & Zhang, 2012 ▸) to generate MR search models based on the sequence of the target structure. *ROSETTA* is also used by *mr_rosetta* (Terwilliger *et al.*, 2012 ▸) from the *PHENIX* suite (Adams *et al.*, 2010 ▸) to rebuild MR solutions that are otherwise difficult to refine and improve upon. *mr_rosetta* can also be used to edit and rebuild search models prior to MR and can assist *Phaser* in scoring MR solutions. Another development designed to tackle these difficult cases is *ARCIMBOLDO* (Rodríguez *et al.*, 2009 ▸), where MR search models are derived from fragment libraries generated from common structural motifs such as helices and β-sheets (Sammito *et al.*, 2013 ▸) or from the systematic shredding of distant homologues (Sammito *et al.*, 2014 ▸). *FRAGON* (Jenkins, 2018 ▸) also uses fragment libraries, rapidly processing them in *Phaser* and then attempting to improve the resulting phases with *ACORN* (Foadi *et al.*, 2000 ▸; Yao *et al.*, 2005 ▸). Another developing trend in unconventional methods is the brute-force trialling of a library of all (or a nonredundant subset) of the set of known structures in MR against a target. This approach requires a large amount of computing power and has been implemented as a grid-based service through the SBGrid Wide Research Molecular Replacement service (Stokes-Rees & Sliz, 2010 ▸) using *Phaser* to perform the MR trials. *CCP*4 distributes an application to perform a similar task, *SIMBAD* (Simpkin *et al.*, 2017 ▸), which uses the rotation-search step in *AMoRe* to rank all of the entries in the nonredundant PDB domain database distributed with the *MoRDa* pipeline against the target data set. Some recent structural studies have also exploited this approach (Hatti *et al.*, 2016 ▸, 2017 ▸; Keegan *et al.*, 2016 ▸).

**Figure 1:**
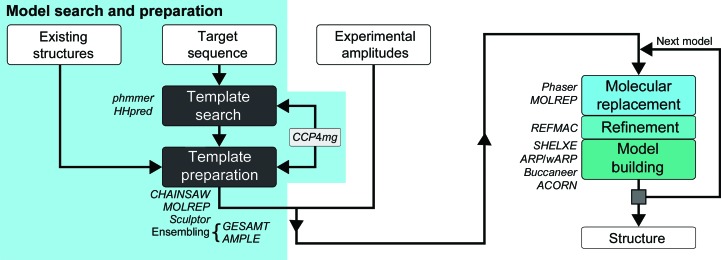
A simple flowchart representation of the *MrBUMP* pipeline. The program can be run in two modes: model search and preparation only or model search, preparation, MR, refinement and model building.

The *MrBUMP* pipeline was originally developed as part of the e-HTPX project (high-throughput protein crystallography; Allan *et al.*, 2005 ▸) and sought to automate phasing through MR as part of a larger project exploring the possibility of automating an MX experiment from expression and purification of the target through to deposition of the final coordinates. *MrBUMP* (Fig. 1 ▸) is now included in the *CCP*4 suite and its scope covers everything from the sourcing and preparation of suitable search models right through to rebuilding of the positioned model. Recent improvements to the pipeline (Table 1 ▸) include the adoption of more sensitive bioinformatic tools for sourcing search models, enhanced model-preparation techniques including better ensembling of homologues, and the use of phase improvement and model building on the resulting solution from MR. The pipeline has also been deployed as an online service through *CCP*4 *online*.

As shown in Fig. 1 ▸, *MrBUMP* has two main modes of operation. The program can be run in a fully automated way from model search through to model rebuilding, or it can be terminated after the model-search and model-preparation steps have completed. The latter mode is useful in cases where a user wishes to carry out MR directly with *Phaser*, *MOLREP* or another MR application using the models generated by *MrBUMP*. The steps taken in this mode are also very quick, taking less than a minute on a single core for a typical target sequence. To enhance the usability of this feature, we combined it with the ability to inspect and modify the produced search models graphically using the *CCP*4*mg* molecular-graphics application (McNicholas *et al.*, 2011 ▸). *CCP*4*mg* was originally developed as a tool to quickly and easily produce publication-quality images as well as movies for presentation purposes. It has a comprehensive range of drawing styles that can be manipulated to aid the interpretation of the structure(s) of interest. Its functionality includes the ability to superpose structures using *SSM*/*Superpose* (Krissinel & Henrick, 2004 ▸) and *GESAMT* (Krissinel, 2012 ▸; Krissinel & Uski, 2017 ▸), as well as providing graphical interfaces to *PISA* (Krissinel & Henrick, 2007 ▸) for exploring macromolecular interfaces and assemblies and to *ProSMART* (Nicholls *et al.*, 2012 ▸) for conformation-independent structural comparison of protein chains. The addition of its interaction with *MrBUMP* further integrates *CCP*4*mg* with some of the core procedures in structure solution and has helped to improve the *CCP*4 user experience in the new *CCP*4*i*2 graphical interface (Potterton *et al.*, 2018 ▸).

One of the recent additions to the pipeline has been the expansion of its use of ensemble search models. In MR, the coordinates of a search model are converted to a calculated structure-factor set for comparison with the experimental data. It has been known for a while that MR search models clustered and aligned into ensembles can be more effective than the individual models themselves (Leahy *et al.*, 1992 ▸; Pieper *et al.*, 1998 ▸; Chen *et al.*, 2000 ▸; Rigden *et al.*, 2002 ▸; Bibby *et al.*, 2012 ▸; Keegan *et al.*, 2015 ▸). *Phaser* can exploit this alignment to produce a statistically weighted structure-factor set based on the variance across the aligned models (Read, 2001 ▸). This helps to improve the signal to noise in its maximum-likelihood function and also assists in performing the packing function (McCoy *et al.*, 2007 ▸). Similarly to *Phaser*, *MOLREP* can also exploit ensembling of search models in two different ways: by calculating structure factors from the entire ensemble or by treating each member of the ensemble as a separate search model. In *MrBUMP*, the former is used when processing ensemble models with *MOLREP*. An additional benefit of ensembling homologues is that the alignment can be used as a guide for truncation. This has been demonstrated by the *AMPLE* project (Bibby *et al.*, 2012 ▸), in which *ab initio* ‘decoy’ models are generated for use in MR. The ensembling of many decoys and sampling the resulting ensembles at many levels of truncation based on the variance of aligned C^α^-atom positions can help to remove parts of the decoy models that are not conserved in the target, an approach inspired by the work of Qian *et al.* (2007 ▸) and Rigden *et al.* (2008 ▸). In turn, this enhances the chances of correctly placing the ensemble model in MR. Based on the experience of solving the structure of angiotensinogen (Zhou *et al.*, 2010 ▸), *sculpt_ensemble* from *PHENIX* (Adams *et al.*, 2010 ▸) implements a similar approach using superposed homologues and a trimming threshold based on the r.m.s.d. between the superposed models.

For single or ensemble search models, *Phaser* weights the structure-factor set from the model using the estimated r.m.s.d. between the coordinates of the model and those of the target structure. An initial value for the r.m.s.d. can be estimated from the sequence identity of the search model to the target. Internally, *Phaser* will adjust this initial estimate of the r.m.s.d. using its variance-r.m.s. (VRMS) parameter to optimize the calculation of its log-likelihood gain score (LLG) and to enhance the chances of picking out the correct solution (Oeffner *et al.*, 2013 ▸). In *Phaser* v.2.7.17, new methodology (R. J. Read, personal communication) was added to determine whether the level of structure-factor agreement among structure factors computed from the models within an ensemble is consistent with the r.m.s.d. estimates provided by the user. In cases where the user’s estimates are too low to be consistent, more appropriate values are derived from the computed structure factors of the ensemble.

## 2. The *MrBUMP* pipeline implementation   

### 2.1. Finding a suitable search model for MR   

Apart from the scenario where an existing structure of the target is available, the first step in conventional MR is to identify a suitable homologue or set of homologues, usually by a sequence-based search against the PDB. The result of the alignment of a sequence against a database of sequences will differ according to the program and accompanying parameters used. In the context of MR, it is an attempt to infer structural similarity from the amino-acid sequence similarity. Fortunately for crystallographers, the field of bioinformatics has seen the development of sensitive sequence-alignment tools designed to exploit the explosion of sequence information being produced by the genomics field. *MrBUMP* now has the option to use two such programs: *phmmer* and *HHpred*.

#### 2.1.1. *phmmer*   

*phmmer* is part of the *HMMER* suite of programs (Eddy, 2011 ▸) designed to search sequence databases for sequence homologues. Its methods use probabilistic models called hidden Markov models (HMMs) to help to find distant homologues for a given sequence profile or individual sequence. Similarly to the *BLAST* *(Basic Local Alignment Search Tool*) application (Altschul *et al.*, 1990 ▸), it searches using a single protein query sequence against a database of sequences.

*phmmer* is distributed with the *CCP*4 suite and is now used (in preference to the original *FASTA*; Pearson & Lipman, 1988 ▸) as the default method in *MrBUMP* for identifying homologues of the target for use as search models. *MrBUMP* includes a curated database of sequences from known protein structures for use in the *phmmer* search. Currently, this database contains entries from the PDB up to 2016. Updates to the database are made available through the *CCP*4 update system on an infrequent basis. Work is being performed to automate the database generation and allow updates to be made with every *CCP*4 update release. A particular advance in the present version of *MrBUMP* is the implementation of several versions of the database, each with a different level of redundancy removed from the complete list of sequences. The redundancy-level options are 100 (no redundancy removed), 95, 90, 70 and 50%. For example, in the 90% redundant set, where two or more structures with a sequence identity greater than 90% are present in the complete list only one example is retained. The sequence lists are drawn from those provided by the PDB (https://www.rcsb.org/pdb/statistics/clusterStatistics.do), which are created using the *BLASTClust* program from the *BLAST* package (Altschul *et al.*, 1990 ▸). When removing similar sequences, the sequence with the highest resolution and the lowest *R* value is preferred.

The addition of these nonredundant sequence lists is one of the advances that were introduced as a result of the inspection of models in the *CCP*4*mg* implementation (§[Sec sec3]3) and is a significant advance on the original implementation, which could only use the 100% set. The redundancy default is now set to 95%, enabling a broad sampling of search models to be trialled in MR, and avoids the pipeline choosing a set of essentially identical search models, such as depositions for the same protein with a series of ligands bound. Use of the latter results in a restricted survey of possible models, where all of the top ten may be essentially identical. However, sequence similarity does not always correspond to structural similarity, so in some cases it can help to use the 100% option and retain structures with very similar sequences: this can be particularly useful for a protein that can adopt a range of different conformations through hinge movements, for example. It may also help to use the 100% option when creating ensemble search models, where small variations in the alignment between a set of very similar structures can guide the identification of the most conserved core residues. This will be discussed in more detail in §[Sec sec2.2.2]2.2.2. His tags and sequences shorter than ten residues are removed from the databases so as to prevent search results being dominated by false-positive matches or short-fragment models.

The target sequence provided to *MrBUMP* is used as input to *phmmer*, which will return a set of matches with scores based on how similar they are to the target sequence. These scores are based on the sequence identity as well as other factors such as gap-opening and gap-extension penalties, and substitution, insertion and deletion scores. To save time in preparation and in MR, a cutoff score of 20 is used to eliminate unrelated proteins and homologues that may be too dissimilar for use as search models. However, this value can be adjusted through the *MrBUMP* PMRCUTOFF keyword when using the program through *CCP*4*i* or from the command line. *phmmer* produces a multiple sequence alignment of the target sequence and those of all of the homologues. The pairwise alignments are extracted from the *phmmer* alignment and are converted by *MrBUMP* into FASTA- and PIR-formatted alignment files. These are retained for use as a guide for the truncation of the corresponding search models in preparation for their use in MR. More details of search-model preparation in *MrBUMP* are given below and in previous publications (Keegan & Winn, 2008 ▸).

#### 2.1.2. Clustering search models for ensemble generation with *phmmer*   

**Figure 2:**
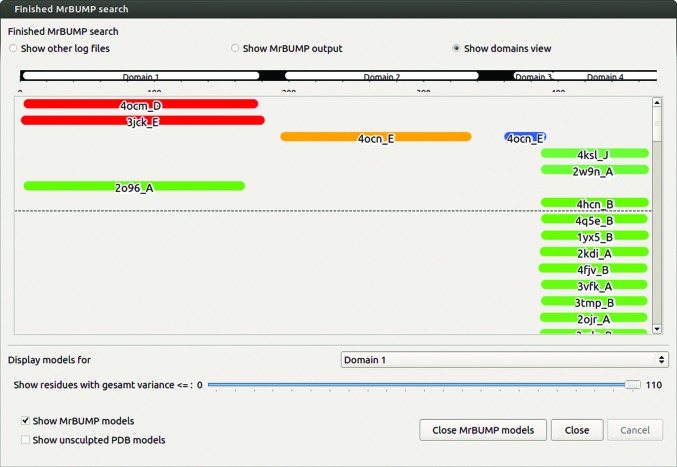
The *CCP*4*mg* interface to *MrBUMP*, with an illustrative representation of the domain regions found during the clustering of matches from the *phmmer* search. The results shown here are for a search using the sequence from PDB entry 5u4p, a protein–protein complex between 26S proteasome regulatory subunit RPN8, RPN11 and ubiquitin S31 (Worden *et al.*, 2017 ▸). The results have been clustered into four domains, two of which consist of more than one match, making them suitable for ensemble generation. The dashed line indicates the cutoff *phmmer* score (default = 20) used for the selection of matches to be used as search models in MR.

Ensembles are derived from an alignment-truncation procedure using the homologues found in the sequence-based search. A detailed description of ensemble generation is provided in §[Sec sec2.2.2]2.2.2. The sequence search may yield a selection of matches to different parts of the target sequence. To construct useful ensembles, it is important to group or cluster homologues based on their structural similarity. As a simple method of achieving this, we have inferred this similarity from the *phmmer* multiple sequence alignment. The midpoint of each aligned sequence in the target-sequence numbering along with the extent that it covers in terms of number of residues in the target is used to decide the cluster that it belongs to. The approach may not be valid in all cases, but we found that clustering sequence matches based on a midpoint tolerance of ±10 residues and an extent tolerance of ±25 residues produces clusters that are suitable for effective ensemble generation. The method is a simplistic attempt at classifying ‘domains’ within the set of sequence matches. We use the word ‘domain’ here to describe a cluster grouping. They may or may not correspond to what are generally considered to be structural or biological domains. When using the *CCP*4*mg* interface, cluster grouping is presented with rainbow colouring of matches based on their *phmmer* score (Fig. 2 ▸). This colour scheme is inspired by the *HHpred* (Söding *et al.*, 2005 ▸) web-application colouring scheme, with red indicating a high-scoring match through to blue at the lower end of the scale. Internally, *MrBUMP* assigns each domain a number and places the corresponding search models in a folder tagged by their domain number. Each group is aligned to create additional ensemble search models. This clustering facilitates the handling of heterogeneous complex targets, which is not yet implemented automatically in *MrBUMP* but can be performed manually when using the *CCP*4*mg*/*MrBUMP* interface *via* *CCP*4*i*2.

#### 2.1.3. *HHpred*   

*phmmer* is quick and does not require the installation of any third-party applications, but it is limited by the fact that when used in *MrBUMP* it only has the set of known PDB sequences from which to derive an alignment. *HHpred* (Söding *et al.*, 2005 ▸; Alva *et al.*, 2016 ▸) is a profile–profile comparison tool based on hidden Markov models, which is often considered to be the most sensitive sequence-comparison method (Söding, 2005 ▸). It can accept a single sequence (used to search a database such as UniProt20) or a multiple alignment as an input profile and can make use of various databases such as the PDB, SCOP (Murzin *et al.*, 1995 ▸), Pfam (Finn *et al.*, 2016 ▸) *etc.* for its search. It is available as a web application through the Max Planck Institute (MPI) Bio­informatics Toolkit (https://toolkit.tuebingen.mpg.de). In difficult MR cases where there are no obvious homologues, its sensitivity to distant relationships between structures can help to find a suitable search model. *MrBUMP* can use *HHpred* if it is installed locally by the user. It requires the installation of the *HHsuite* software suite in addition to the substantial PDB70 and UniProt20 databases. These are available from https://github.com/soedinglab/hh-suite. Currently, this option is only accessible to *MrBUMP* through *CCP*4*i* and *CCP*4 *online*. It will be added to the *CCP*4*i*2 interface in the near future.

*HHpred* also provides a multiple sequence alignment between the target and the matches that it has found in its search. As with the *phmmer* search results, when used in *MrBUMP* the *HHpred* alignments are extracted and saved as pairwise alignment files (FASTA and PIR format) for use in the search-model preparation stage.

### 2.2. Search-model preparation   

The success or failure of molecular replacement is highly sensitive to the similarity between a search model and the target structure (Schwarzenbacher *et al.*, 2004 ▸). Once a search model has been found, it is always best to prepare it in such a way that, based on available knowledge, it is made as similar as possible to the target. The goal is to conserve structural detail that is common to both the model and the target, and remove the nonconserved parts. The information provided by the alignment of the search-model sequence with that of the target can be used as a guide. Side-chain truncation and loop trimming are two examples of how the alignment can be exploited. In addition, structural alignment of a set of homologues can give some indication of the features that are most likely to be present in the target. As described above, MR programs such as *Phaser* can exploit the ensembling of homologues to weight the experimental data based on how much the main chains of the search models vary in their alignment. *MrBUMP* can prepare ensemble models from the list of homologues found in the search step, which are then included in the set of search models to be tested in MR.

#### 2.2.1. Homologue modification   

Several programs within *CCP*4 can carry out homologue modification. *MrBUMP* can utilize each of these for adjustment of the homologues to create MR search models. Where required, the pairwise alignment generated by the sequence-based homology search (*phmmer* or *HHpred*) is used as input to the modification procedure.

(i) *Sculptor*. *Sculptor* (Bunkóczi & Read, 2011 ▸) can be used to generate ‘mixed-model’ search models for MR. It takes in an alignment between a target sequence and that of the homologue and the corresponding structure of the homologue. Based on the sequence alignment, it modifies the homologue to create a search model in three different ways: main-chain deletion, side-chain pruning and *B*-factor modification. These options can be used in combination or separately. *MrBUMP* uses the default protocol when using *Sculptor*. It is based on an algorithm outlined in Schwarzenbacher *et al.* (2004 ▸) that includes settings such as pruning non-identical side chains to the C^γ^ atom and using the original *B* factors to predict new *B* factors.
(ii) *CHAINSAW*. *CHAINSAW* (Stein, 2008 ▸) also carries out modifications to a homologue based on a provided sequence alignment between the target and the homologue. It conserves, truncates or deletes residues in the homologue based on the alignment.
(iii) *MOLREP*. *MOLREP* can generate mixed-model search models when provided with the sequences of the target and a homologue. It performs its own internal alignment between the sequence of the target and that of the homologue and truncates the model accordingly (Lebedev *et al.*, 2008 ▸).

Each of these programs produces very similar results, but in difficult cases it is worthwhile trying all of them, as small variations in the model can be the difference between success or failure (Keegan *et al.*, 2011 ▸). In addition, *MrBUMP* has some other homologue-modification options. These include reducing the search model to a polyalanine backbone and retaining the homologue unmodified. The latter can be useful in situations where a user has prepared a search model or a set of search models to be fed into *MrBUMP*.

#### 2.2.2. Ensembles   

*MrBUMP* can generate ensemble models from the homologues that it finds during the search step. It aligns the resulting search models based on the domain group to which they have been assigned (currently only by *phmmer*). These ensembles are then put through a truncation procedure which produces a set of derived ensemble models that can be added to the list of search models to be processed in MR. Within the pipeline, there are now two new approaches to ensembling and truncating homologues. These involve the use of the *GESAMT* or *AMPLE* programs, which are also provided through the *CCP*4 suite. Both programs facilitate the generation of truncated ensembles based on the variance between the aligned search models.

(i) *GESAMT*. *GESAMT* (*General Efficient Structural Alignment of Macromolecular Targets*) is a recently developed structural alignment application. It improves upon previous alignment applications such as *SSM* by better enabling the alignment of fragmented or incomplete models. This was achieved by making the alignment independent of secondary structure. It creates a global alignment by comparing locally similar fragments of the given models. This makes it ideal for comparing the structures of two or more potential MR search models which may only share regions of localized similarity. It produces a table indicating the distances between aligned C^α^-atom positions. Within the model-preparation stage of *MrBUMP*, the selected homologues from the search step are put through *Sculptor* and then passed to *GESAMT* for structural alignment. A base alignment file is produced that contains the full set of residues of all of the aligned structures in a single PDB file. Truncation is applied to this base alignment to produce several derived ensembles. By default, 20 derived ensembles are generated, with each being produced by truncating the most variable parts of the base alignment in steps of 5%. The most variable will contain 100% of the base alignment residues, while the least variable retains only the most conserved 5% of the base alignment residues. If several domains are found in the *phmmer* search, the set of search models for each domain will be aligned and their corresponding set of truncated ensembles will be generated.
(ii) *AMPLE*. *AMPLE* is a *CCP*4 development that was initially designed to exploit the rapidly developing field of *ab initio* modelling of protein structures for use as search models in MR. Using sequence information alone, programs such as *ROSETTA* and *QUARK* can generate approximations to the target structure, which are known as ‘decoys’. For a given target, *AMPLE* uses these programs to generate hundreds of such decoy models and attempts to use them as search models in MR. Key to its approach is a cluster-truncation procedure for preparing the decoys in an attempt to isolate regions that are also present in the target. Internally, *AMPLE* uses the maximum-likelihood superposition method in *THESEUS* (Theobold & Wuttke, 2008 ▸; Theobald & Steindel, 2012 ▸) to perform the alignment between decoys. We have found that alignment in *THESEUS* can give a lower variance between the aligned C^α^-atom positions in the core region of the structures compared with alignment in *GESAMT*. This provides an alternative set of truncated ensemble search models. We have adapted the cluster-truncation procedure of *AMPLE* in *MrBUMP* to cluster and truncate the set of homologues that it finds in its sequence search.*AMPLE* passes its truncated ensemble models to *Phaser* for MR. Producing these models using *ab initio* techniques breaks the sequence identity–structural similarity relationship. All search models are created to have the target sequence, but may vary widely in their structural similarity to the target. This makes it difficult to gauge the correct values to input to *Phaser* for the search-model r.m.s.d. The adopted strategy was to assume that these models had an extremely low r.m.s.d. to the target and use a value of 0.1 Å. In testing using the version of *Phaser* (v.2.5.4) available at the time, this value was found to work well with these models, particularly for small-fragment models consisting of 10–20 residues (Keegan *et al.*, 2015 ▸). It enabled *Phaser* to give more weight to the high-resolution reflections and gave these small, yet highly accurate search models the best chance of being placed correctly. As discussed above, a recent update to *Phaser* allows it to generate a more appropriate r.m.s.d. based on the internal r.m.s.d. of a provided ensemble. *AMPLE* now exploits this method of parameterization for the r.m.s.d. of its generated ensembles when using *Phaser* to perform MR. As part of this work, we sought to understand the interaction between ensemble truncation and the r.m.s.d. value provided to *Phaser*. This study will be presented in the example case in §[Sec sec5]5.

### 2.3. Molecular replacement in *MrBUMP*   

*MrBUMP* uses *Phaser* and/or *MOLREP* to carry out the molecular-replacement step. Both programs are highly automated and default parameters are used for most of their options. The only program options set by *MrBUMP* are the anticipated number of molecules expected in the asymmetric unit, which is calculated using *MATTHEWS_COEF* (Matthews, 1968 ▸; Kantardjieff & Rupp, 2003 ▸), and the expected r.m.s.d. value for each search model given to *Phaser*.

### 2.4. Determining whether a molecular-replacement result is correct   

#### 2.4.1. MR scoring   

Both *Phaser* and *MOLREP* provide scoring systems that can give a good indication as to whether or not a search model has been placed correctly in MR. The *Phaser* LLG (log-likelihood-gain) score (Storoni *et al.*, 2004 ▸; McCoy *et al.*, 2005 ▸; Oeffner *et al.*, 2013 ▸; Read & McCoy, 2016 ▸) is the most reliable indicator of the correctness of its solutions. An increase of 60 in the LLG on the placement of a new molecule is a strong indication of success (McCoy *et al.*, 2017 ▸). Values below 60 can still correspond to a correct solution but require further examination. A single, standout solution is also a good indication of success. As a general rule, the *Phaser* scoring system is sensitive to the accuracy of what is specified as the composition of the asymmetric unit of the target and the estimated r.m.s.d. from the target structure assigned to the input model. Users running *Phaser* directly are advised to pay close attention to these details. *MOLREP* does not use maximum likelihood in its scoring, but presents the user with *Z*-scores for the strength of the peaks in the rotation and translation searches. Typically, values for the TF/σ of above 8 indicate correct placement, but values below this can still represent a correct solution. A clear, standout value for the top peak in the RF/σ and TF/σ scores is usually the strongest indication of success. If several copies are expected in the asymmetric unit there may be several strong peaks present in these scores.

#### 2.4.2. Refinement of the MR solution   

Refining the positioned search model from MR can help to further validate the correctness of the solution. Within *MrBUMP*, *REFMAC* (Murshudov *et al.*, 2011 ▸) is used to refine the output model from MR, employing a default of 100 cycles of restrained refinement applying ‘jelly-body’ restraints with defaults used for the harmonic distance restraints in the refinement target function (RIDG DIST SIGM 0.02). This compares with the 30 cycles of restrained refinement employed in the original version of *MrBUMP*. The use of jelly-body restraints, which stabilize a refinement by modifying the curvature of the target function, is particularly useful after MR and can help with solutions from distant homology search models where large fragments of the model can deviate significantly from the correct position.

*MrBUMP* categorizes MR solutions based on the *R*_free_ values after refinement (Keegan & Winn, 2008 ▸). Solutions are classed as ‘GOOD’ (*R*_free_ < 0.35), ‘MARGINAL’ (*R*_free_ < 0.5) or ‘POOR’ (*R*_free_ > 0.5) based on the behaviour of *R*_free_, but this categorization is quite conservative and even poor solutions should be examined in further detail if nothing better is produced. As the job progresses, a summary table of the scores for all search models that have completed their trial in MR is produced. This table is sorted according to final *R*_free_ values.

#### 2.4.3. Phase improvement and model building   

The final stage of the pipeline can now optionally perform phase improvement and model (re-)building using the refined search model as a starting point for the phases of the target. The main purpose is to further validate the MR solution, but with the benefit of producing a model for the target structure that is more suitable for subsequent manual model building and refinement. There are several program options that can be selected within the *MrBUMP* pipeline, namely *SHELXE* (Thorn & Sheldrick, 2013 ▸; Usón & Sheldrick, 2018 ▸), *ARP*/*wARP* (Langer *et al.*, 2008 ▸) and *Buccaneer* (Cowtan, 2012 ▸).

(i) *SHELXE*. Where the resolution of the experimental data permits (typically better than 2.4 Å), a run of *SHELXE* will, by default, be invoked to attempt density modification followed by polyalanine tracing into the improved electron-density map. The initial set of phases for the target are derived by *SHELXE* from the refined MR solution model produced by *REFMAC* in the refinement step of *MrBUMP*. By default, 15 global cycles of density modification (20 iterations per global cycle) and polyalanine tracing are invoked. The resulting polyalanine model after each cycle is used as a new estimate for the phases of the target for the next cycle. Other parameters passed to *SHELXE* by *MrBUMP* include the -q option to search for α-helices, the -o option to optimize the CC of the input model, the -s option to provide the estimated solvent fraction and the -e1.0 option to add missing ‘free-lunch’ data up to 1.0 Å resolution when the observed data resolution extends to 2.0 Å or better. Given favourable circumstances (for example a resolution of ∼2.4 Å or better), it has been shown (Thorn & Sheldrick, 2013 ▸; Keegan *et al.*, 2015 ▸) that a correlation coefficient (CC) of greater than 25% between the native structure factors and those calculated from the polyalanine trace, combined with an average traced chain length of ten residues or more, is a reliable indication of a correctly traced model. This in turn indicates that the positioning of the search model by MR was correct. *SHELXE* can be particularly useful in building upon an MR solution where only a small but accurate fragment model has been used as a search model and placed correctly. This has been demonstrated extensively by both the *AMPLE* and *ARCIMBOLDO* programs.
(ii) *Buccaneer* and *ARP*/*wARP*. *SHELXE* is primarily a density-modification program with polyalanine tracing. *Buccaneer* and *ARP*/*wARP* are specifically designed for model building into an electron-density map. Both require that the phases used to generate the map are already approximately correct and can improve upon a correctly placed MR search model, bringing its structure closer to that of the expected target. Their metrics for a correct model are the *R*/*R*_free_ values from refinement with *REFMAC* after each cycle of model building. A good solution from either program is a clear indication of the success of MR and can provide a better starting model for further model building and refinement than the original MR solution. In the *MrBUMP* pipeline, options allow these programs to be run immediately after the refinement step, using the refined MR model as a starting point for the phase information, or after *SHELXE*, using its polyalanine trace model as the starting model. This second option is particularly useful for removing model bias at resolutions better than 2.4 Å.
(iii) *ACORN*. *ACORN* is a program for phase improvement using dynamic density modification and can be invoked through the USEAcorn keyword in *MrBUMP*. It is not currently invoked by default or made available through the interfaces owing to the overlap of its functionality with that of *SHELXE*, which is less restrictive in the required resolution for the observed reflection data (the recommeded low-resolution limit for *ACORN* is 1.7 Å; Yao *et al.*, 2005 ▸). Output phases from *ACORN* are not currently passed on to the model-building steps in *MrBUMP*. A detailed description of the use of *ACORN* in *MrBUMP* can be found in Keegan & Winn (2008 ▸).

### 2.5. *MrBUMP* output   

Ensemble models are first tested in MR. Upon completion, they are followed by the single-chain search models according to how they scored in the *phmmer*/*HHpred* results. The output of the pipeline is an ongoing summary of the various scores for the completed MR trials. The summary is presented as a table of search models ranked according to the final *R*_free_ value after refinement of the positioned MR model. *Phaser* scores and model-building results are also presented in the table. The paths to the various PDB and MTZ files as well as log files for the current top-scoring model are presented, and a final summary table is produced when the job completes. All of the resulting files and logs for each of the MR trials can be found in the ‘data’ subdirectory of the top-level *MrBUMP* directory (denoted by search_‘*job identifier*’). The set of created search models are placed in the ‘models’ subdirectory. This directory will contain domain subdirectories, each having an additional ensembles directory containing the set of generated ensemble PDB files.

## 3. *MrBUMP* through *CCP*4*mg*   

Having the capability to view and modify search models can be useful in difficult cases. Such cases might include, for example, instances where the homologous structures found by *phmmer* have multiple domains and no structural alignment can adequately superpose all of the domains. For such proteins the user may wish to specify which domain to use as the search model, or to use more than one domain with different structural alignments. In other instances, only distant homologues may be found and it may be necessary to use only part of the structures in MR.

Usage of this option can be performed directly from *CCP*4*mg* or from the Bioinformatics task menu in the *CCP*4*i*2 graphical interface. With a user-supplied sequence and a chosen nonredundancy level for the homologue search, *MrBUMP* uses *phmmer* to search the PDB sequence database and download homologous structures. These are then prepared for MR using *Sculptor* and structurally aligned with *GESAMT*, with the resulting structural alignment being displayed in the *CCP*4*mg* graphical window. *CCP*4*mg* will also display the sequence alignment, as described in §[Sec sec2.1.1]2.1.1 and shown in Fig. 2 ▸. The residues displayed in *CCP*4*mg* are tagged with the spatial variance of the C^α^ atoms of the superposed structures as reported by *GESAMT*, and a slider can be used to control the display of more or less structurally variable residues. In this way, the user can select and save their choice of a well defined ‘core’ structure to use in MR. The user is furthermore free to use the whole range of atom-selection tools in *CCP*4*mg* (specific chains, residues, atoms, neighbourhoods *etc.*) to choose what to draw and save as a search model.

By default, the largest domain common to the structures as defined by *phmmer* is displayed. The results window allows the user to show and hide any of the found domains. When a domain is chosen, the ‘GESAMT variance slider’ is provided to allow the selection of atoms based on the structural alignment of that domain. It is alternatively possible to display and select the models before the *Sculptor* step of *MrBUMP*.

When satisfied with the displayed atoms, the user saves them to an ensemble PDB file suitable for input to *Phaser* or *MOLREP*. A normal PDB file can in addition be saved for each individual structure. When using the *CCP*4*mg*/*MrBUMP* procedure through the *CCP*4*i*2 graphical interface, the recommended action is to save the files to the *CCP*4*i*2 database for seamless integration with the other tasks. The full set of log files produced by *MrBUMP* can be inspected from within *CCP*4*mg* by clicking on the appropriate button in the results window (Fig. 2 ▸).

The integration of *MrBUMP* and *CCP*4*mg* not only creates a mechanism for attempting to produce successful MR models where pure automation fails, but also acts as a powerful aid to the developers of the pipeline in helping to understand how changes to the model search and preparation stages affect the resulting search models. Indeed it has already led to several of the advances described here, such as selection of the appropriate sequence-redundancy level or of alternative structural domains.

## 4. *MrBUMP* web application   

*CCP*4 *online* (Krissinel *et al.*, 2018 ▸) is a web-based portal facilitating the execution of compute-intensive components of the *CCP*4 suite on the *CCP*4 compute clusters based at the Research Complex at Harwell (RCaH), and *MrBUMP* is one of the services it provides. The deployment of the service in this way has several advantages to the user and the developers. The processing of each search model in MR, refinement and model building is farmed out to the cluster, allowing more rapid calculation of results. The service also uses the *HHpred* search tools and databases to identify potential search models. The search programs in *HHpred* require large databases of tens of gigabytes in size, which are large to download and are not always practical for local installation. The other advantage is that developers can deploy the latest stable versions on the centralized installation, removing the need for users to keep their local installations up to date.

Since its launch in 2014, the service has been heavily used, with, for example, 1721 *MrBUMP* jobs run in 2016. A total of 1204 users have registered to use the service. User data are not retained beyond five months, and detailed statistics on success and failure rates have only been collected for the five months prior to the time of writing. This covers the period September 2017 to January 2018. Within this period, 788 jobs were initiated, of which 497 completed, with the remainder terminating early for various reasons. Determining success or failure for the completed jobs is difficult without examining each result manually. User data are treated as confidential, so we can only obtain a general overview of the success rate based on the scoring statistics from the various programs in the pipeline. Table 2 ▸ presents some statistics for what is usually considered a successful solution from the 497 completed jobs. The input data resolution ranged from 1.0 to 7.4 Å.

Of the jobs that terminated early, 201 failed as a result of missing requirements in the input data, for example no FreeR column or unrecognized formatting of the sequence file. Other jobs which terminated early included 19 where the predicted unit-cell content was too large for the given unit-cell parameters and 12 where no matches to the target sequence could be found in the *HHpred* search.

All data and log files for each program in the pipeline are made available to the user to download as they are produced. A zipped tarball containing all files is made available at the end of the job. The version of *MrBUMP* and the underlying programs that it uses are dependent on the version of *CCP*4 installed on the server. This is updated regularly. At the time of writing, *CCP*4 v.7.0.050 is being utilized. To access the *MrBUMP* web application, please visit http://www.ccp4.ac.uk/ccp4online.

## 5. Exploring the solution for PDB entry 5cml using *CCP*4*mg*/*MrBUMP*   

As discussed in §[Sec sec3]3, the *CCP*4*mg* interface to *MrBUMP* allows a user to view and modify the set of search models found and prepared by the pipeline. The large set of model-selection and model-manipulation tools available in *CCP*4*mg*, including the ability to truncate the ensembled search models, allow a user to generate any number of permutations of the original model set. For example, a user may choose to truncate away the most variable 10% of the ensemble using the slider tool and save the reduced ensemble as a search model for MR. Alternatively, they could select only the highest scoring member of the reduced ensemble and use that as a search model. To evaluate how best to use the capabilities of this method of running *MrBUMP*, we explored its use for the case of PDB entry 5cml, a protein-domain structure from the bacterium *Rhodothermus marinus* (Jensen *et al.*, 2016 ▸). We chose this example as it has the characteristics of a difficult case for MR. When originally solved, the only homologues available had sequence identities in the range 20–30%. This put it in the boundary zone of where sequence identity can be relied upon to be indicative of structural similarity. The original solution required the generation of an ensemble search model derived from PDB entries 2fuk, 3trd and 3pf9. Here, we attempted to solve it again using the *CCP*4*mg*/*MrBUMP* application. We explored the use of both single and ensemble search models using several degrees of truncation to establish what the optimum choice of search-model edits in *CCP*4*mg* would be for this case. There are two copies in the asymmetric unit to search for, each consisting of 263 residues. The space group is *P*2_1_, with unit-cell parameters *a* = 60.33, *b* = 74.07, *c* = 60.95 Å, α = 90.00, β = 113.47, γ = 90.00°. The resolution of the experimental data is 1.56 Å and the solvent content is 42.85%.

By default, when run through *CCP*4*i*2, a *Phaser* run using search models from *CCP*4*mg*/*MrBUMP* will take the r.m.s.d. information for the search model from the *GESAMT* alignment, which is contained in the search-model PDB file REMARK cards. Each member of the ensemble will have a corresponding r.m.s.d. from a centroid structure. Where a single search model is derived from the initial ensemble, it will carry its corresponding r.m.s.d. value from the original alignment through to *Phaser*. Alternatively, users can provide a sequence identity or an r.m.s.d. which has been produced from some other source such as a sequence alignment. To understand how the choice of r.m.s.d. provided to *Phaser* influences the final solution when using models created through the *CCP*4*mg*/*MrBUMP* application, we looked at what happened when it was varied across a wide range of values for the 5cml case. We performed this study using several levels of truncation for both ensemble and single search models. The true r.m.s.d. between the target and the search models used ranged from 0.4 to 1.8 Å depending on the degree of truncation applied to the model. In the case of the ensemble search models, the true r.m.s.d. varies across the members of the ensemble at each truncation level. For simplicity, we set their input r.m.s.d. to be the same. The range used here was from 0.1 to 2.4 Å in steps of 0.1 Å. Using r.m.s.d values as low as 0.1 Å makes little sense for nontruncated homologues or for ensembles where the internal r.m.s.d. exceeds this value, but can be useful for small-fragment search models that very closely match fragments in the target. We also examined how the steps in *MrBUMP* following MR, specifically refinement with *REFMAC* and density modification and polyalanine tracing with *SHELXE*, helped to improve upon the initial MR solution and also assist in assessing its correctness.

### 5.1. Search-model selection   

**Figure 3:**
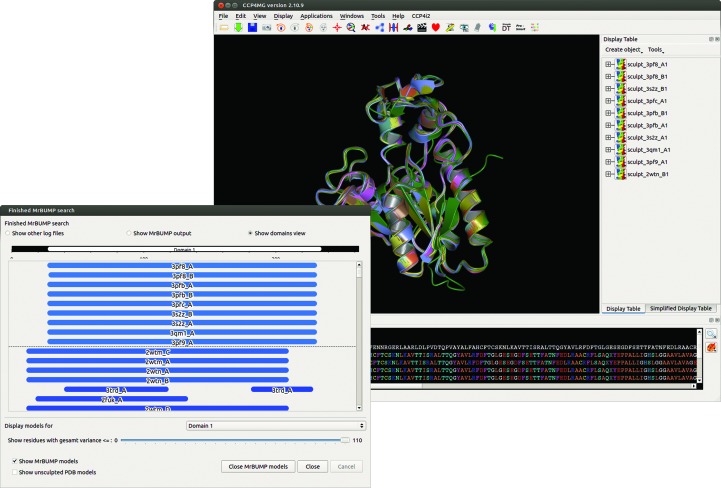
The *CCP*4*mg*/*MrBUMP* interface displaying the results of a search using the sequence from PDB entry 5cml. Search models found in the search step are pruned using *Sculptor* and aligned using the *GESAMT* structure-alignment program. The resulting ensemble consists of ten individual search models derived from the following chains: chains *A* and *B* of PDB entries 3pf8, 3pfb and 3s2z, chain *A* of PDB entries 3pfc, 3qm1 and 3pf9, and chain *B* of PDB entry 2wtn.

To simulate a novel structure solution, we used a reduced version of the PDB only containing entries that have accession codes commencing with 1, 2, 3 and 4. This corresponds to a separation of several months between the most recent entry that can be used as a search model and the time of deposition for PDB entry 5cml (deposited in July 2015). We used the full database of sequences (no redundancy removal) from the reduced version of the PDB in the homologue-search step. The best homologue found in the *phmmer* search was chain *A* of PDB entry 3pf8 (deposited in October 2010), with a *phmmer* score of 53.9. According to a *PSI-BLAST* search, this structure has a low sequence identity of 26% to the target with a query coverage of 78%, values that are indicative of a challenging problem for MR. An additional nine structures (Fig. 3 ▸), including chain *B* of PDB entry 3pf8, scored similarly and exhibited very similar folds when aligned structurally. Table 3 ▸ shows the sequence identity (according to *PSI-BLAST*), the pairwise aligned r.m.s.d. against chain *A* of PDB entry 5cml (using *GESAMT*) and the the *phmmer* score for each of the ten structures. A multiple alignment of all ten using *GESAMT* gives a mean r.m.s.d. of 0.38 Å from a calculated centroid for the aligned C^α^-atom positions.

### 5.2. Ensemble preparation   

Fig. 3 ▸ displays the *CCP*4*mg* graphical window with a ribbon representation of the ten search models after *Sculptor* and aligned using *GESAMT*. This is referred to as the ‘base’ ensemble before any truncation has been applied. As described in §[Sec sec3]3, *GESAMT* applies tags to aligned residues that are considered ‘core’ to the alignment. These core residues are the 100% truncation level. The base ensemble may contain additional residues outside this core in some or all of the incorporated models. For convenience, the base ensemble is referred to as 110% in the *CCP*4*mg* interface, although it may constitute more or less content than this implies. Here, the base ensemble consists of models ranging in length from 197 to 204 residues. The *GESAMT* core has 184 residues in all of the incorporated models.

**Figure 4:**
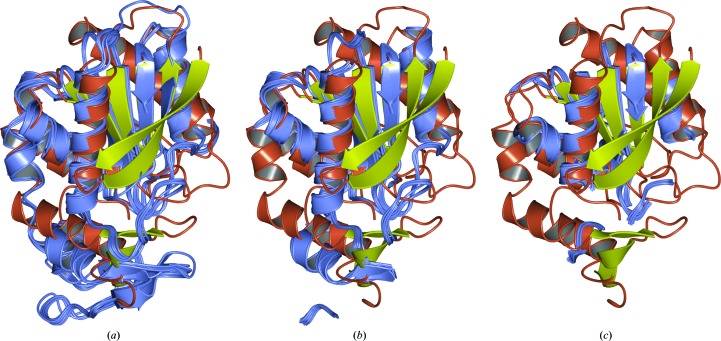
Three levels of the truncated ensemble model (ice blue) aligned with chain *A* of PDB entry 5cml (orange/yellow). (*a*) Base ensemble, (*b*) 80% truncation level, (*c*) 40% truncation level.

To demonstrate the effectiveness of ensembling and truncating the search models, we produced ten derived ensembles from the initial base ensemble. These models were created using the truncation slider tool in the *CCP*4*mg* graphical window, moving it in steps of 10% and exporting the displayed coordinates to a PDB file. Note that the same set of search models can be produced when running *MrBUMP* in its automated mode. Each of these ensembles is a reduced version of the base, with a C^α^-atom position variance tolerance ranging from 10 to 100% of the *GESAMT* core residues. The base ensemble, as well as ensembles truncated at the 80 and 40% levels, are shown in Fig. 4 ▸. For the sake of comparison with a single-model search in MR, we extracted the components deriving from the highest scoring homologue (chain *A* of PDB entry 3pf8) from each of the 11 ensembles and used them as additional search models. It is possible that some of the other members of the ensemble may yield a solution where chain *A* of PDB entry 3pf8 does not. However, we wish to demonstrate that producing an ensemble search model from the full set of homologues can be more effective in MR than an individual component search model on its own.

### 5.3. Molecular replacement   

Each of these ensembles, including the base ensemble, along with the corresponding single models, were used as MR search models for the 5cml target using *Phaser* (v.2.7.17). We instructed *Phaser* to search for two copies of the search model. Each model was tested in *Phaser* at each of the r.m.s.d. settings between 0.1 and 2.4 Å. A total of 496 individual tests were run using the CCP4 Linux cluster at the RCaH: 24 values for the input *Phaser* r.m.s.d. applied to 11 truncation levels (including the base level) for both the ensemble and the single search models.

### 5.4. Assessing the MR solution   

Each solution from *Phaser* was put through 100 cycles of restrained refinement using jelly-body restraints in *REFMAC* (v.5.8.0155). The resulting refined model was input to *SHELXE* (v.2018/1) to provide initial phases for 15 cycles of density modification and polyalanine tracing. To assess the correctness of the solution at each step, we calculated the map correlation coefficient (mapCC) between the electron density calculated from the deposited experimental data and structure for 5cml and a calculated map after the *Phaser*, *REFMAC* and *SHELXE* steps (Fig. 6). The mapCC calculations were performed using *phenix.get_cc_mtz_mtz* from the *PHENIX* suite (Adams *et al.*, 2010 ▸).

## 6. Results   

**Figure 5:**
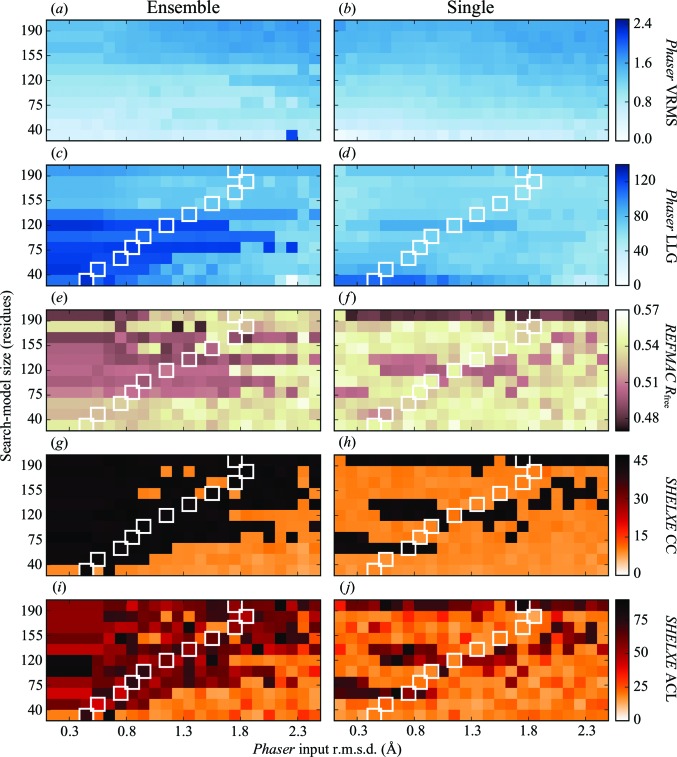
Colour maps of various scores and values of the input *Phaser* r.m.s.d. against the truncation level for ensemble and single models. Results are displayed for the final variance-r.m.s. (VRMS) value from *Phaser* (*a*, *b*), the final log-likelihood gain (LLG) scores from *Phaser* (*c*, *d*), the final *R*_free_ value from *REFMAC* after 100 cycles using jelly-body restraints (*e*, *f*), the *SHELXE* CC between the native structure factors and those calculated from the output polyalanine trace model (*g*, *h*) and the *SHELXE* output polyalanine trace model average chain length (ACL) (*i*, *j*). As a reference, the white boxes show the r.m.s.d. estimate calculated by pairwise alignment in *GESAMT* for the single search model against the target structure at each truncation level.

Various values and scores are presented for the 496 test runs in Fig. 5 ▸. These include the VRMS calculated by *Phaser* (Figs. 5 ▸ *a* and 5 ▸ *b*), the final log-likelihood gain (LLG) score for the top solution from *Phaser* (Figs. 5 ▸ *c* and 5 ▸ *d*), the final *R*_free_ value after 100 cycles of restrained refinement using jelly-body restraints in *REFMAC* (Figs. 5 ▸ *e* and 5 ▸ *f*), the *SHELXE* correlation coefficient (CC) between the native data and the polyalanine trace that it produces (Figs. 5 ▸ *g* and 5 ▸ *h*), and the average chain length (ACL) for the polyalanine trace produced by *SHELXE* (Figs. 5 ▸ *i* and 5 ▸ *j*). These results are discussed in detail in the following sections.

### 6.1. Phaser VRMS   

**Figure 6:**
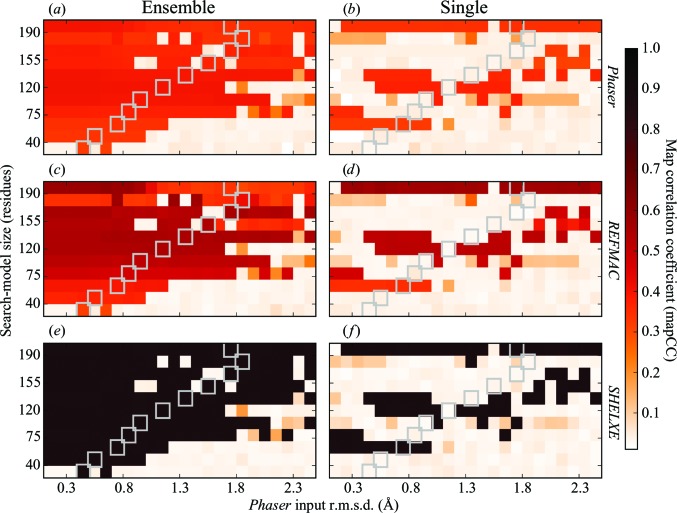
The map correlation coefficient (mapCC) for the electron-density maps generated after the *Phaser* (*a*, *b*), *REFMAC* (*c*, *d*) and *SHELXE* (*e*, *f*) steps compared with a map calculated from the deposited intensities and structure for PDB entry 5cml. Results are shown for all input *Phaser* r.m.s.d. values and all truncation levels (ensemble and single search models). The colour plots illustrate how the map CC increases after each step for most of the correctly placed MR solutions. The map coefficients generated by both *Phaser* and *REFMAC* were used in the comparison. The deposited amplitudes were used in combination with the calculated phases from *SHELXE* to generate a map for the *SHELXE* comparison. As a reference, the grey boxes show the r.m.s.d. estimate calculated by pairwise alignment in *GESAMT* for the single search model against the target at each truncation level.

Figs. 5 ▸(*a*) and 5 ▸(*b*) show a colour map of the calculated VRMS value produced by *Phaser* for the 248 ensemble and single tests, respectively. For the cases where a search model produces a correct solution, the associated VRMS values are seen to be refined to a value close to the true r.m.s.d. between the search model and the target structure. In these cases the final VRMS is almost independent of the initial r.m.s.d. estimate, but in general it does depend on the initial estimate *via* the probability of finding the correct solution, as can be seen by comparison of Figs. 5 ▸(*a*) and 5 ▸(*b*) with Fig. 6 ▸ (for incorrect or partially incorrect solutions the refined values of VRMS are higher).

As the level of truncation increases, the VRMS decreases, reflecting the increasing structural similarity of the search model to the target structure. For the ensemble search models, values typically range from 1.7 Å for the base ensemble to 0.4 Å for the 10% truncation level (19 residues). For the single models these values varied from 1.7 to 0.15 Å.

### 6.2. Phaser log-likelihood gain (LLG)   

The final LLG scores from *Phaser* are plotted in Figs. 5 ▸(*c*) and 5 ▸(*d*) for the ensemble and single search-model tests. For both search-model types the optimum truncation level was at 40–70% (75–135 residues), although higher levels of truncation combined with low r.m.s.d. values also show high LLG scores. In these ranges, LLG values of up to 127 (ensemble) and 110 (single) are reported. These were mostly single standout solutions, well separated in score from alternative positions, indicating a good chance of success. Values for LLG are also notably high for the most truncated search models (ensemble and single) where low r.m.s.d. values are used. Indeed, at 0.4 and 0.6 Å r.m.s.d. for the 10% truncated ensemble search model the LLG reaches 127 and 121, respectively, indicating correct placement. The high scores for these two cases can be explained by the fact that the r.m.s.d. values are close to the correct value (as shown by the box in Fig. 5 ▸ *c*). With accurate parameterization, *Phaser* is capable of finding the correct placement for such small search models (McCoy *et al.*, 2017 ▸). Furthermore, these cases have amongst the lowest mean phase errors with respect to the correct phases (79.8°) of any of the positioned search models. These two cases go on to produce definitive solutions, as shown in the mapCC plots (Fig. 6 ▸). For the single-model tests, the 10% truncated search model reaches an LLG of 112 for several of the provided r.m.s.d. values; however, no solution is obtained from any of these cases.

The nontruncated search model in the ensemble tests gives LLG values of between 83.0 and 93.0 depending on the r.m.s.d. provided. Although all tests with this search model resulted in correct placement, refinement of the placed solutions (Fig. 5 ▸ *e*) reveals that the initial placement accuracy of the model is sensitive to the r.m.s.d. given to *Phaser* and ultimately affects the ease with which jelly-body refinement can improve upon the solution. Greater accuracy results in lower *R* factors and better density (Fig. 6 ▸ *c*). The same effect is not observed when using the single nontruncated search model (Fig. 5 ▸ *f*).

### 6.3. *REFMAC* final *R*_free_   

The final *R*_free_ values from 100 cycles of restrained refinement with jelly-body restraints are presented in Figs. 5 ▸(*e*) and 5 ▸(*f*). The benefits of refining the MR solutions are made clear in the single-model tests (Fig. 5 ▸ *f*), where using the original nontruncated search model and an input r.m.s.d. for *Phaser* in the range 0.2–2.4 Å (with the exception of 1.5 Å) results in *R*_free_ values between 0.47 and 0.5. The mean mapCC value for these solutions (Fig. 6 ▸ *d*) is 0.57, up from 0.34 after the *Phaser* step. Use of the single-chain model (chain *A* of PDB entry 3pf8) worked on its own and leads to clear solutions in the *SHELXE* step. It is possible that recent improvements in *Phaser*, such as the introduction of the VRMS or using *Sculptor* to prepare the search model, have made it possible to solve the case with this single chain rather than having to use an ensemble as was performed in the original structure solution of PDB entry 5cml. Overall, 63 ensemble cases and 26 single-model cases achieve an *R*_free_ of lower than 0.5.

### 6.4. *SHELXE* CC and polyalanine trace average chain length   

Figs. 5 ▸(*g*) and 5 ▸(*h*) show the CC values from *SHELXE* for the ensemble and single tests. Figs. 5 ▸(*i*) and 5 ▸(*j*) show the corresponding average chain length (ACL) for the *SHELXE* polyalanine trace. We have found that owing to changes to *SHELXE* implemented in the version (2018/1) used in this study, an ACL of greater than 10 cannot be taken as a reliable indicator of success, as had been observed with previous versions of the program.

In a novel case, we could use the *SHELXE* success criteria as an indicator for success in MR. These scores are typically only reliable for resolutions better than 2.5 Å, but we will compare them with the mapCC results (Figs. 6 ▸ *e* and 6 ▸ *f*) to see how well they predict success in this case. Based on a CC of ≥25, we find that the ensemble search models have produced 186/248 solutions, with the single search models producing 69/248 solutions. Without exception, the successes based on a *SHELXE* CC of ≥25 correlate directly with results with high mapCC (≥0.88). With few exceptions, results with ACL ≥ 35 correlate with the same set of mapCC results.

It is worth noting that in Fig. 5 ▸(*i*) the ACL values at the lowest r.m.s.d. values are identical at each truncation level (between 0.4 and 0.6 Å depending on the degree of truncation). In these cases, *Phaser* has parameterized the r.m.s.d. of the ensemble search models based on that generated by the alignment of the members of the ensemble, supplanting that provided as input. As discussed in §[Sec sec1]1, this is performed to correct the impossible scenario in which the r.m.s.d. to the target structure is assumed to be lower than the internal alignment of the ensemble.

### 6.5. Map correlation coefficient   

Results for the mapCC calculations are presented in Fig. 6 ▸ for the ensemble and single test cases. For the overall majority of correct solutions, the mapCC is seen to increase after each step, illustrating the benefit of refining and rebuilding the MR solution as is performed within *MrBUMP*. Using *Solution_Check* from the *CCP*4 suite (Winn *et al.*, 2011 ▸), we examined those cases that show a low but not insignificant mapCC (between 0.1 and 0.25). These cases (seven ensemble cases and 18 single cases) were found to be solutions in which only the first of the two copies of the search model had been placed correctly, with the second being incorrectly placed. These fail to achieve an improvement in the mapCC as they pass through the *REFMAC* and *SHELXE* steps.

## 7. Conclusions   

The *MrBUMP* automated pipeline for molecular replacement has been significantly enhanced in recent years, with improvements in its search-model selection and preparation steps, and post-MR refinement, as well as the use of density modification and automated model building to improve the positioned search model. Taken together, these changes improve the performance, user-friendliness and success indicators of the software. Its integration with the *CCP*4*mg* molecular-graphics program provides a valuable tool for the visual examination and manipulation of search models for MR. Users can also benefit from using *MrBUMP* through the *CCP*4 *online* web application. Advantages of the latter include access to the *HHpred* software and databases for a more sensitive detection of potential search models and a manyfold speedup through the distribution of search-model processing in MR onto a compute cluster.

In exploring the solution to PDB entry 5cml, comparison of the ensemble and single mapCC plots (Fig. 6 ▸) reveals that when using the *CCP*4*mg*/*MrBUMP* tool to select a search model for use in *Phaser*, the original ensemble or a truncated derivative of it is more likely to succeed than selecting the chain with the highest sequence identity from the ensemble at an equivalent truncation level. Truncation of the search model also works better for ensembles, with solutions being produced for ensembles even at the 10% truncation level (19 residues), provided that an accurate estimate of the r.m.s.d. between the search model and the target is used. As described in Read (2001 ▸) and Oeffner *et al.* (2013 ▸), an accurate estimation of the r.m.s.d. is important for calibration of the likelihood function in *Phaser*. This is illustrated in the both the ensemble and single search-model tests: solutions across the range of r.m.s.d. values used in the tests correlate well with how the truncation of the search model lowers the true r.m.s.d. against the target structure.

Future improvements to *MrBUMP* will include the ability to input the original experimental intensity measurements rather than the calculated structure-factor amplitudes. This is to match the default in *Phaser*, where intensities are used in preference to amplitudes to account better for measurement error in the experiment in its likelihood functions (Read & McCoy, 2016 ▸). Other enhancements will include the automatic provision of updates to the *phmmer* sequence databases and the deployment of *MrBUMP* through the *CCP*4 Cloud development *jsCoFE* (JavaScript-powered Cloud Front End) described by Krissinel *et al.* (2018 ▸). This has the potential to eventually replace *CCP*4 *online*, giving users the ability to run *MrBUMP* within a project-based environment online, rather than as a standalone application.

## 8. Availability   

*MrBUMP* and *CCP*4*mg* are distributed under the CCP4 licence and are included in the *CCP*4 suite, which is available for download from http://www.ccp4.ac.uk. *MrBUMP* runs under Linux/Unix, Mac OSX and Windows, and comes with *CCP*4*i* and *CCP*4*i*2 interfaces. The *MrBUMP* web application is available from http://www.ccp4.ac.uk/ccp4online. Table 4 ▸ gives a breakdown of the *MrBUMP* protocols and defaults used for the various *CCP*4 platforms.

## Figures and Tables

**Table 1:** Summary of changes to the *MrBUMP* pipeline since the 2011 version

*MrBUMP* stages	2011 version	2017 version
Model search	Sequence-based search (*FASTA*)	Sequence-based search, domain identification and alignment generation (*phmmer*, *HHpred*)
	Domain identification (SCOP)	
	Alignment generation (*ClustalW*, *MAFFT*)	
Search-model preparation	Generation of mixed models based on sequence alignment (*CHAINSAW*, *MOLREP*)	Graphical search-model interaction (*CCP*4*mg*)
	Single untruncated ensemble (*Superpose*)	Generation of mixed models based on sequence alignment (*Sculptor*, *CHAINSAW*, *MOLREP*)
		Multiple truncated ensembles (using *GESAMT* or *AMPLE*)
Molecular replacement	MR (*Phaser*, *MOLREP*)	MR (*Phaser*, *MOLREP*)
Refinement	30 cycles of restrained refinement (*REFMAC*)	100 cycles of restrained refinement with jelly-body restraints (*REFMAC*)
Density modification	Phase improvement for target data better than 1.7 Å resolution (*ACORN*)	Phase improvement for target data better than 2.4 Å (*SHELXE*) or 1.7 Å (*ACORN*) resolution
Model building		Polyalanine main-chain tracing (*SHELXE*)
		Full model building (*ARP*/*wARP* and *Buccaneer*)

**Table 2:** Statistics from 497 recently completed jobs run on the *MrBUMP* web service Results are separated for input data resolutions better and worse than 2.5 Å. Solutions that achieve the stated scores tend to overlap; each row represents a subset of the row above. However, there are several cases where *SHELXE* achieves a CC of >25 where the scoring criteria for other programs have not been achieved, particularly when the data resolution is ≤2.5 Å.

	No. of user jobs achieving this score			
	Data resolution ≤ 2.5 Å (264 jobs)		Data resolution > 2.5 Å (233 jobs)	
	*Phaser*	*MOLREP*	*Phaser*	*MOLREP*
LLG ≥ 60	144	N/A	103	N/A
TFZ ≥ 8	120	N/A	87	N/A
*REFMAC* final *R*_free_ ≤ 0.5	97	81	67	72
*Buccaneer* build final *R*_free_ ≤ 0.5	87	79	42	31
*SHELXE* CC ≥ 25 and ACL ≥ 10	68	62	4	3

**Table 3:** *phmmer* search results for the sequence of PDB entry 5cml Sequence-identity details are taken from *PSI-BLAST* results for the same sequence. The calculated r.m.s.d. from *GESAMT* for the core residues from a pairwise alignment with the structure of PDB entry 5cml is also presented. The multiple alignment of these ten models is the base alignment for ensemble generation. (Note that the final character in each model name is a domain identifier.)

	*PSI-BLAST *sequence identity (%)	R.m.s.d. against PDB entry 5cml (Å)	*Phmmer* score
3pf8_*A*1	26	1.735	53.9
3pf8_*B*1	26	1.790	53.5
3pf9_*A*1	25	1.780	52.5
3pfb_*A*1	25	1.722	52.6
3pfb_*B*1	25	1.715	52.6
3pfc_*A*1	25	1.775	52.6
3qm1_*A*1	25	1.781	52.5
3s2z_*A*1	25	1.739	52.5
3s2z_*B*1	25	1.750	52.6
2wtn_*B*1	24	1.739	43.9

**Table 4:** Availability of *MrBUMP* through the various *CCP*4 interfaces *CCP*4*i* provides fine-grained control of the program for all steps, while the *CCP*4*i*2 implementation is meant to be quick and more automated, requiring minimal input from the user, and *CCP*4*mg* gives an interactive and graphical view of the model-preparation step. *CCP*4 *online* has the advantage of being able to make use of large databases for the *HHpred* search and compute clusters for parallel processing of search models.

	*CCP*4*i*	*CCP*4*i*2	*CCP*4*mg*	*CCP*4 *online*
Automated model search and MR	Yes	Yes		Yes
Customized model search and preparation	Yes	Yes (*via* *CCP*4*mg*)	Yes	
Customized MR and model building	Yes			
Default search method	*Phmmer*	*Phmmer*	*Phmmer*	*HHpred*
Default preparation methods	*Sculptor*	*Sculptor*	*Sculptor *	*Sculptor*
	*MOLREP*	*GESAMT*	*GESAMT*	*MOLREP*
	*CHAINSAW*			*CHAINSAW*
Default MR programs	*Phaser*	*Phaser*	N/A	*Phaser*
	*MOLREP*			*MOLREP*
Default refinement cycles	100 (jelly body)	100 (jelly body)	N/A	100 (jelly body)
Default model-building programs	*SHELXE* (resolution < 2.4 Å)	None	N/A	*SHELXE *(resolution < 2.4 Å)
	*Buccaneer*			*Buccaneer*
*CCP*4 version (as of 5 February 2018)	*CCP*4 7.0.050	*CCP*4 7.0.050	*CCP*4 7.0.050	*CCP*4 7.0.050
